# Mortality and survival in nonagenarians during the COVID-19 pandemic: Unstable equilibrium of aging

**DOI:** 10.3389/fmed.2023.1132476

**Published:** 2023-03-02

**Authors:** Daria A. Kashtanova, Veronika V. Erema, Maria S. Gusakova, Ekaterina R. Sutulova, Anna Yu. Yakovchik, Mikhail V. Ivanov, Anastasiia N. Taraskina, Mikhail V. Terekhov, Lorena R. Matkava, Antonina M. Rumyantseva, Vladimir S. Yudin, Anna A. Akopyan, Irina D. Strazhesko, Irina S. Kordiukova, Alexandra I. Akinshina, Valentin V. Makarov, Olga N. Tkacheva, Sergey A. Kraevoy, Sergey M. Yudin

**Affiliations:** ^1^Federal State Budgetary Institution “Centre for Strategic Planning and Management of Biomedical Health Risks” of the Federal Medical Biological Agency, Moscow, Russia; ^2^Russian Clinical Research Center for Gerontology, Pirogov Russian National Research Medical University, Ministry of Healthcare of the Russian Federation, Moscow, Russia

**Keywords:** inflammaging, mortality, nonagenarians, COVID-19, longevity, aging, COVID-19 mortality, mortality predictors

## Abstract

**Introduction:**

Aging puts the human body under an immense stress and makes it extremely susceptible to many diseases, often leading to poor outcomes and even death. Long-living individuals represent a unique group of people who withstood the stress of time and offer an abundance of information on the body’s ability to endure the pressure of aging. In this study, we sought to identify predictors of overall one-year mortality in 1641 long-living individuals. Additionally, we analyzed risk factors for COVID-19-related morality, since statistics demonstrated an extreme vulnerability of older adults.

**Methods:**

We conducted a two-stage evaluation, including a comprehensive geriatric assessment for major aging-associated: frailty, cognitive impairment, frontal lobe dysfunction, chronic pain, anxiety, risk of falls, sensory deficit, depression, sarcopenia, risk of malnutrition, fecal and urinary incontinence, dependence in Activities of Daily Living, dependence in Instrumental Activities of Daily Living, polypragmasia, and orthostatic hypotension; extensive blood testing, a survey, and a one-year follow-up interview.

**Results:**

The most reliable predictors of overall mortality were cognitive impairment, malnutrition, frailty, aging-associated diseases and blood markers indicating malnutrition-induced metabolic dysfunctions (decreased levels of protein fractions, iron, 25-hydroxyvitamin D, and HDL), and aging biomarkers, such as IGF-1 and N-terminal pro b-type natriuretic peptide. In post-COVID 19 participants, the most significant mortality predictors among geriatric syndromes were depression, frontal lobe dysfunction and frailty, and similar to overall mortality blood biomarkers - 25-hydroxyvitamin D, IGF-1, HDL as well as high white blood cell, neutrophils counts and proinflammatory markers. Based on the results, we built a predictive model of overall mortality in the long-living individuals with f-score=0.76.

**Conclusion:**

The most sensitive and reliable predictors of mortality were modifiable. This is another evidence of the critical importance of proper geriatric care and support for individuals in their “golden years”. These results could facilitate geriatric institutions in their pursuit for providing improved care and could aid physicians in detecting early signs of potentially deadly outcomes. Additionally, our findings could be used in developing day-to-day care guidelines, which would greatly improve prevention statistics.

## Introduction

Globally, people are living longer, and the number of older adults is increasing. Aging causes significant changes in the human body and results in numerous chronic conditions, which make the older population progressively more susceptible to diseases and poor outcomes (1, 2). This has been unequivocally demonstrated by the COVID-19 pandemic. Counterintuitively, aging also causes some of the risk factors to gain – protective properties. Identifying predictors of poor outcomes in older adults would contribute to timely disease prevention and provide a better understanding of the physiology of aging. Sadly, older adults are too often excluded from relevant studies, the findings of which could directly affect their wellbeing. Here, we focused on long-living adults who represent a unique aging phenotype. A number of studies have addressed mortality in long-living adults has been addressed in (3–5). The Danish 1905-Cohort Survey investigated factors associated with mortality in over 2000 participants aged 85 years and above. The authors found that the 15-month mortality was associated with the degree of disability; a low level of mental and physical activity; and, in women, low self-esteem (6). In the PLAD study of mortality in a Chinese cohort of the oldest-old, age and aging-associated diseases were predictors of mortality, whereas high MMSE scores and high level of physical activity contributed to survival; moreover, the survival rate was higher in women (7). The Mugello Study found that cognitive disorders, ADL, polypragmasy, and renal dysfunction were predictors of the 12-month mortality in nonagenarians (8).

During the COVID-19 pandemic, several studies analyzed the COVID-19-related mortality in people older than 90 years of age. The mortality rate was higher in the 90-year-old hospitalized patients with functional dependencies (1). Analysis of mortality rates in people aged 60 years and above showed that dementia was associated with an increased COVID-19-related mortality rate (9).

These used data from cohorts from different countries and projects and have constantly revealed common patterns, which significantly contribute to mortality. They were mostly focused on non-modifiable markers reflective of current health status. However, prevention of undesirable outcomes largely relies on predictive and modifiable markers.

In the present study, we analyzed data from 1,641 long-living Russian adults aged 90 years and above. We assessed their health status and examined associations between geriatric syndromes (GSs), clinical and biochemical parameters and one-year mortality. Presently, this study is the largest of this design type in Russia. Coincidentally, it was carried out during the COVID-19 pandemic; therefore, we investigated the association between the studied factors and COVID-19-related mortality.

## Methods

### Study design

Initially, we recruited 2020 long-living adults aged 90 years and above. To provide validity, we based our results on full sets of data, which were collected in a two-stage procedure. Data sets for 379 participants were incomplete at the end of the second stage; therefore, we excluded them from the study, which brought the number of participants down to 1,641.

All participants provided informed consent.

The first stage was conducted in 2020: participants were visited by a physician and a nurse for a comprehensive geriatric assessment, biomaterial sampling (whole blood and blood serum), and survey completion. The survey aimed at collecting information about health status, assessing lifelong risks of chronic diseases, and analyzing the lifestyle and socioeconomic background. The comprehensive geriatric assessment focused on the following GSs (Table 1) and followed the Clinical guidelines on frailty, approved by the Ministry of Health of the Russian Federation (10). Supplementary Table S1 provides more information about GS assessment:

**Table 1:** Geriatric syndromes and methods of their assessment.

Geriatric syndrome	Assessment method
Frailty	The short physical performance battery (SPPB)
Cognitive impairment	The mini-mental state examination (MMSE)
Frontal lobe dysfunction	The frontal assessment battery (FAB)
Chronic pain	Questionnaire 1 (provided in Supplementary Table S1: Questionnaires)
Anxiety	Questionnaire 2 (provided in Supplementary Table S1: Questionnaires)
Risk of falls	Questionnaire 3 (provided in Supplementary Table S1: Questionnaires)
Sensory deficit	Questionnaire 4 (provided in Supplementary Table S1: Questionnaires)
Depression	The five-item geriatric depression scale (GDS 5)
Sarcopenia	The Simple questionnaire to rapidly diagnose sarcopenia (SARC-F)
Risk of malnutrition	The mini nutritional assessment (MNA)
Fecal and urinary incontinence	The Barthel Index
Activities of daily living (ADLs)	The Barthel Index
Instrumental activities of daily living (IADLs)	The Lawton scale
Polypragmasia	Interpreted as a simultaneous administration of five or more medications
Orthostatic hypotension	Blood pressure test (sitting vs. standing)

The blood and serum samples were tested for:

- Complete blood count and white blood cell differential (with Sysmex hematology analyzers).
- Glucose metabolic panel (glycosylated hemoglobin (colorimetric analysis), glucose (hexokinase analysis) and insulin (immunoassay) levels).
- Lipid panel (cholesterol (enzymatic analysis), triglycerides (homogeneous enzymatic colorimetric assay), low-density lipoprotein [a direct measurement method for colorimetric determination of cholesterol oxidase and cholesterol esterase) and high-density lipoprotein levels (homogeneous enzymatic colorimetric assay)]; 4. Hepatic cytolysis markers (ALT and AST), Bilirubin and gamma-GT.
- Markers of hepatocyte cytolysis (ALT and AST) (kinetic UV method), bilirubin [colorimetric assay with a diazo reagent (Endrashik method)], GGT (kinetic colorimetric assay).
- Ferritin (enzyme-immunoassay), homocysteine (enzyme-immunoassay), fibrinogen (Clauss Method: by thrombin clotting time in diluted plasma).
- Uric acid [enzymatic (uricase).
- Renal function test: creatinine (enzymatic), urea (kinetic UV method (urease)) and cystatin C levels (immunoturbidimetry).
- Prostate-Specific Antigen (PSA) (chemiluminescent immunoassay analysis).
- Protein fractions: albumin and globulin (capillary electrophoresis).

Hormonal screening for sex hormones: testosterone (solid phase chemiluminescent immunoassay analysis), estrogen (solid phase chemiluminescent immunoassay analysis) and dehydroepiandrosterone sulfate (enzyme-immunoassay); thyroid hormones: thyroid-stimulating hormone (chemiluminescent immunoassay analysis), free T3 (enzyme-immunoassay), and adipokines: adiponectin and leptin (solid phase enzyme-immunoassay)).

Insulin-like growth factor (enzyme-immunoassay).

N-terminal pro–brain natriuretic peptide as a marker of age-related cardiovascular diseases (electrochemiluminescence immunoassay).

25-hydroxyvitamin D (chemiluminescent microparticle immunoassay).

The second stage was conducted a year later: the participanrs were interviewed by phone to collect up-to-date data on their overall health status, past diseases, Covid-19 status (vaccination status, date of contraction, duration, and severity), and vital status (including, when appropriate, the date and cause of death).

Complete data sets were obtained for 1,641 participants, 347 of whom had recovered and 113 had died from COVID-19.

The present longitudinal observational cohort study is a joint effort by the Centre for Strategic Planning and Management of Biomedical Health Risks and Pirogov’s Russian Clinical and Research Center of Gerontology of the Federal Medical Biological Agency. The study is approved by the Local ethics Committee of the Pirogov’s Russian Clinical and Research Center of Gerontology (Protocol 30 from 24 December, 2019).

### Statistical analysis

For statistical analysis, we used Statsmodels, a Python (v.3.6.9) module. The Shapiro–Wilk test showed that most data were distributed non-normally; therefore, we applied a Box-Cox data transformation. For data description, we used the median and interquartile range (IQR). The categorical variables are expressed as numbers and percentages.

To establish statistically significant associations between complete blood count and blood chemistry and mortality, we performed logistic regression analysis. We used the least squares method (Statsmodels, Python 3.8.) to estimate the model parameters. To measure the importance of the independent variables and calculate the value of ps, we used an F-test. Age and sex were used as covariates.

We used the following function: y = β_1 * x_1 + β_2 * x_2 + β_3 * x_3 + β_0:

y—mortality (1—the number of participants who died within a year after the examination; 0—the number of surviving participants); x1, x2 and x3—sex, age, and the factors, respectively. To establish statistically significant associations between the GSs and mortality, we performed logistic regression analysis (Statsmodels v0.12.2, Python 3.8.). The results are presented as odds ratio (OR), the logistic regression coefficient, and the Pearson’s correlation coefficient.

To decide whether to accept or reject the null hypothesis, we applied the Bonferroni correction.

To build a prognostic model, we used the Random forests algorithm in Scikit-learn, a software machine learning library for the Python programming language, and the data on the tests results, aging-associated diseases, and mortality. We split the data into the training set (80%) and the tests set (20%). The training set was first standardized using the StandardScaler (Scikit-learn). To evaluate the model, we used the ROC-curve and K-fold cross-validation. To calculate the confidence interval for the ROC curve and AUC, we used bootstrap percentile re-sampling with 100 re-samples per model. CI for the ROC curve and AUC was 95%.

As a result, we identified variables significant associated with mortality.

## Results

Out of 1,641 participants, 603 (36.7%) were home-based; 538 (32.8%) resided in elderly care facilities; 500 (30.5%) were inpatients. Vital status (dead or alive) was available for 1,641 participants (out of 2020 initially enrolled); 75% of 1,641 them were women, 32.1% of whom lived alone. The excluded participants (n = 379) did not exhibit any significant differences from other long-living individuals in the given parameters. i.e., gender, age, inclusion criteria, and clinical profiles.

**Figure 1:**
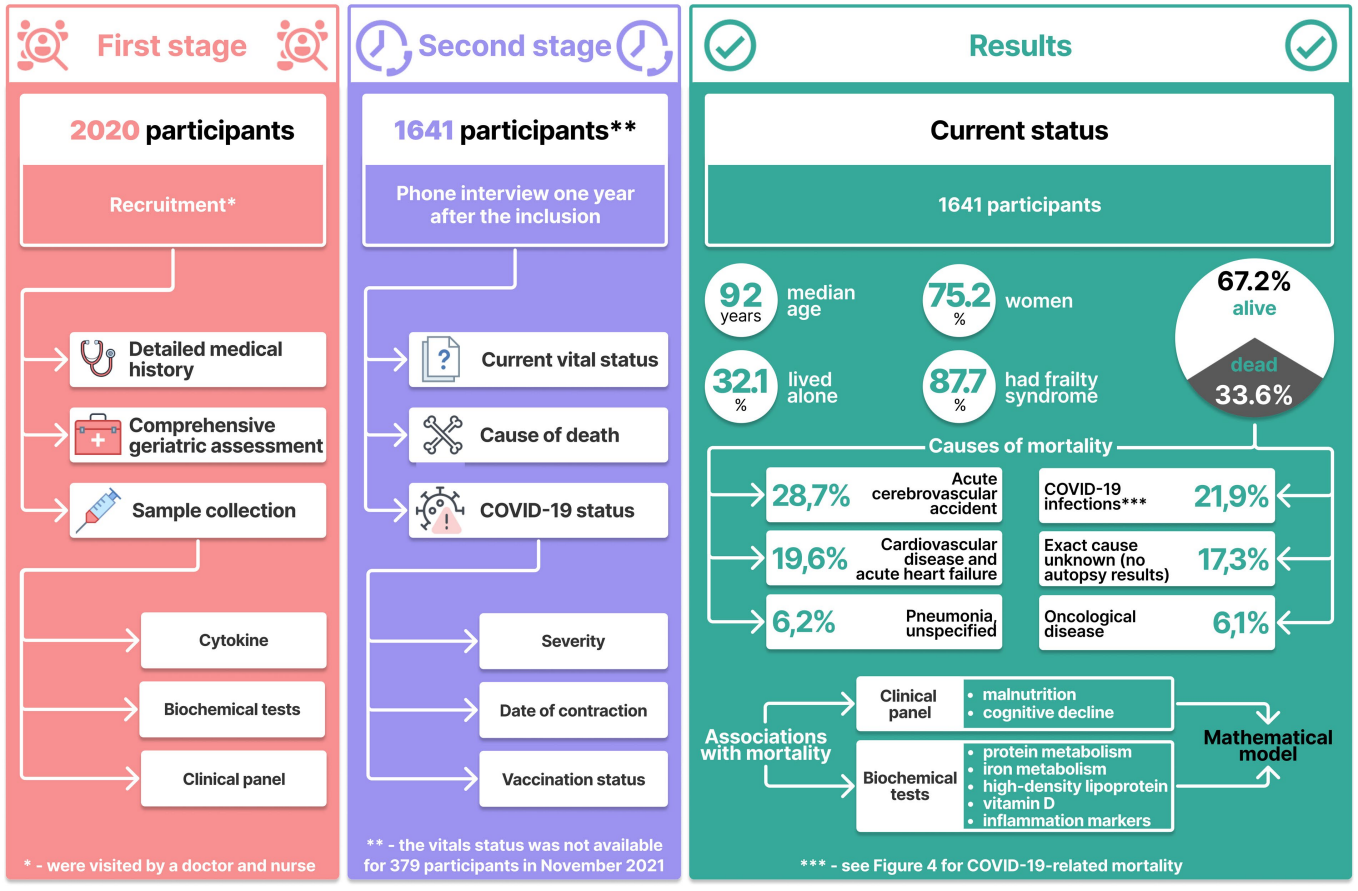
Study design and key results.

The age median was 92 years (Q1-Q3 91–94 years). By late 2021, 552 (33.62%) participants died. Figure 1 shows the participant inclusion algorithm and key examination results.

By 2021, 33.6% (*n* = 552) of the participants (*n* = 1,641) had died. Table 2 shows characteristics of the participants.

**Table 2:** Baseline participant characteristics.

	*N*^†^	Results: median [Q1; Q3] or n (%)
Age in years, median [Q1; Q3]	1,641	92 (91; 94)
Women, n (%)	1,641	1,234 (75.2%)
BMI, kg/m^2^, median [Q1; Q3]	1,503	25.5 (22.9; 28.6)
Living alone, n (%)	1,633	525 (32.1%)
Current smoking status, n (%)	1,579	6 (0.4%)
SPPB score, median [Q1; Q3]	1,537	3 (1; 6)
Frailty (SPPB≤7), n (%)	1,639	1,438 (87.7%)
MMSE score, median [Q1; Q3]	1,542	23 (17; 26)
Cognitive impairment (diagnosed by a neurologist; other than mild cognitive impairment), n (%)	1,542	826 (53.6%)
FAB score, median [Q1; Q3]	1,523	11 (7; 16)
Frontal lobe dysfunction, n (%)	1,564	1,198 (76.6%)
Dependence in ADL, n (%)	1,593	1,463 (91.8%)
Dependence in IADL, n (%)	1,599	1,511 (94.5%)
Chronic pain, n (%)	1,580	996 (63%)
Anxiety, n (%)	1,018	362 (35.6%)
Risk of falls, n (%)	1,576	917 (58.2%)
Sensory deficit, n (%)	1,585	1,483 (93.6%)
GDS-5 score, median [Q1; Q3]	1,493	1 (0; 3)
Depression, n (%)	1,584	764 (48.2%)
MNA score, median [Q1; Q3]	327	20 (17; 22.5)
No malnutrition/risk of malnutrition/malnutrition, n (%)	1,425	199 (14%) /895 (62.8%) /331 (23.2%)
Urinary incontinence, n (%)	493	335 (73.5%)
Fecal incontinence, n (%)	456	151 (33.1%)
Polypragmasia, n (%)	1,464	719 (49.1%)
Orthostatic hypotension, n (%)	1,053	294 (28%)
Aging-associated diseases, n (%)	1,638	1,570 (95.8%)
Cancer, n (%)	1,606	113 (7%)
Cardiovascular diseases, n (%)	1,611	694 (43.1%)
Diabetes mellitus, n (%)	1,612	231 (14.3%)
COPD, n (%)	1,610	234 (14.5%)
Sarcopenia, n (%)	1,450	1,321 (91.1%)
Participants who died in the course of the study, n (%)	1,641	552 (33.6%)
Participants infected by COVID-19, n (%)	347	21.1%
Participants who died from COVID-19, n (% of those who were infected by COVID-19)	113	32.6%

*N*^†^, the number of participants available for the assessment of the characteristic. ADL, activity of daily-living; BMI, body mass index; COVID-19, coronavirus disease 2019; СОРВ, chronic obstructive pulmonary disease; FAB, frontal assessment battery; GDS, geriatric depression scale; IADL, instrumental activity of daily-living; MMSE, mini-mental state examination; MNA, mini nutritional assessment; SPPB, short physical performance battery.

Most participants were affected by at least one GS at the time of enrollment; 90% of them suffered from frailty. The design of this study did not require random sampling; therefore, we cannot provide a specific estimate of the prevalence of GSs in the older adults in Moscow. Nonetheless, we can safely say that there was a positive correlation between age and the number of GSs in this region (11).

Figure 1 shows that the main causes of death in the older adults were cerebrovascular accidents (28.7%) and cardiovascular diseases (19.6%). Another major cause of mortality was COVID-19. This number, however, could be even higher due to COVID-19 complications, but which might have been classified as resulting from cardiovascular diseases.

### All-cause mortality

We investigated the associations between all-cause mortality and the geriatric assessment and blood test results.

### Associations between the geriatric syndromes and all-cause mortality

We found statistically significant associations between mortality and GSs, including malnutrition, ADL and IADL, frailty, frontal lobe dysfunction, a high risk of falls, depression, cognitive impairment, and aging-related diseases.

Table 3 presents the significant associations between the GSs and mortality, adjusted for sex, age, and multiple testing. See Supplementary Table S2 presents all associations between the GSs and mortality.

**Table 3:** Associations between the GSs and 1-year all-cause mortality (only significant), adjusted for sex, age, and multiple testing.

Age, sex and GS	*N*^†^ (alive/dead)	In alive (n, % from the alive)	In deceased (n, % from the dead)	OR	CC	*P-*value	ROC AUC (95% CI)
Age	1,641 (1,089/552)	92 [91; 94] (mean: 92.51)	92 [91;94] (mean: 92.81)	1.13 [1.02; 1.25]	0.12	0.02	0.50 [0.41, 0.56]
Sex	1,641 (1,089/552)	m: 1089 (257, 23,6%) w: 1089 (832, 76,4%)	m: 552 (150, 27,2%) w: 552 (402, 72,8%)	0.92 [0.83; 1.02]	−0.08	0.11	0.52 [0.48, 0.56]
Depression	1,584 (1,068/516)	484 (45.3%)	280 (54.3%)	1.04 [1.02; 1.06]	0.17	0.001	0.55 [0.49, 0.62]
Malnutrition /risk of malnutrition	1,425 (975/450)	193 (19.8%) 626 (64.2%)	138 (30.7%) 269 (59.8%)	1.07 [1.04; 1.09]	0.3	<0.0001	0.59 [0.53, 0.66]
Cognitive impairment	1,542 (1,031/511)	488 (47.3%)	338 (66.1%)	1.09 [1.06; 1.11]	0.4	<0.0001	0.61 [0.54, 0.65]
Frontal lobe dysfunction	1,564 (1,049/515)	752 (71.7%)	446 (86.6%)	1.08 [1.07; 1.13]	0.4	<0.0001	0.60 [0.53, 0.66]
Dependence in ADL	1,593 (1,061/532)	954 (89.9%)	509 (95.7%)	1.05 [1.03; 1.09]	0.2	<0.0001	0.57 [0.50, 0.63]
Dependence in IADL	1,599 (1,065/534)	988 (92.8%)	523 (97.9%)	1.05 [1.03; 1.10]	0.3	<0.0001	0.56 [0.51, 0.61]
Frailty	1,639 (1,088/551)	922 (84.7%)	516 (93.6%)	1.06 [1.04; 1.10]	0.3	<0.0001	0.57 [0.51, 0.63]
AADs	1,638 (1,087/551)	1,026 (94.4%)	544 (98.7%)	1.05 [1.03; 1.10]	0.3	<0.0001	0.55 [0.49, 0.61]

*N*^†^, the number of participants, available for the assessment of the parameter; CC, correlation coefficient, CI, confidence interval; OR, odds ratio; ROC, receiver operating characteristic curve; AUC, area under curve; AADs, aging-associated diseases; ADL, activity of daily-living; IADL, instrumental activity of daily-living.

Cognitive impairment was the main contributor to mortality in the older adults. It even surpassed frailty, which is conventionally viewed as an unsuccessful aging phenotype, and aging-associated diseases.

### Association between the test results and mortality

The multivariate regression analysis, the results of which were adjusted for sex, age, and multiple testing revealed significant associations between the test results and mortality (Table 4). All associations are presented in Supplementary Table S3.

**Table 4:** Associations (only significant) between the test results and 1-year all-cause mortality, adjusted for sex, age, and multiple testing.

Test	In alive (*n* = 1,089) median [Q1; Q3]	In deceased (*n* = 552) median [Q1; Q3]	OR	CC (normalized)	*p*-value	ROC AUC (95% CI)
Hemoglobin, g/dL	12.6 [11.3; 13.7]	12.2 [11; 13.5]	0.90 [0.85; 0.96] per unit of measure	−0.20	0.0004	0.56 [0.50, 0.62]
МСHС, g/dL	33.2 [32.4; 34]	32.9 [31.9; 33.8]	0.86 [0.80; 0.93] per unit of measure	−0.22	<0.0001	0.58 [0.52, 0.65]
RDW, %	13.9 [13.2; 14.8]	14.25 [13.3; 15.5]	1.13 [1.07; 1.18] per unit of measure	0.27	<0.0001	0.58 [0.51, 0.63]
Total protein, g/L	70 [66; 75]	69 [64; 73]	0.96 [0.95; 0.98] per unit of measure	−0.26	<0.0001	0.57 [0.50, 0.63]
Albumin, g/L	39.2 [35.8; 41.925]	36.8 [33.3; 40]	0.90 [0.88; 0.92] per unit of measure	−0.51	<0.0001	0.64 [0.59, 0.68]
α1-globulin, g/L	3.1 [2.8; 3.4]	3.3 [3; 3.7]	1.83 [1.54; 2.17] per unit of measure	0.39	<0.0001	0.60 [0.54, 0.66]
Total cholesterol, mmol/L	4.93 [4.11; 5.72]	4.72 [3.9; 5.6]	0.86 [0.79; 0.94] per unit of measure	−0.19	0.0007	0.55 [0.50, 0.60]
HDL, mmol/L	1.3 [1.1; 1.6]	1.18 [0.96; 1.4]	0.33 [0.24; 0.45] per unit of measure	−0.40	<0.0001	0.60 [0.56, 0.65]
AC	2.7 [2.1; 3.4]	2.9 [2.2; 3.8]	1.18 [1.08; 1.29] per unit of measure	0.20	0.0001	0.56 [0.51, 0.62]
ALT, U/L	12 [9; 15]	11 [8; 15]	0.98 [0.97; 0.99] per unit of measure	−0.20	0.0001	0.53 [0.47, 0.58]
GGT, U/L	18 [14; 29]	17 [12; 27]	0.97 [0.94; 0.98] per 10 units of measure	−0.18	0.0008	0.54 [0.49, 0.59]
hsCRP, mg/L	2.62 [1.31; 6.77]	3.99 [1.83; 10.71]	1.02 [1.01; 1.02] per unit of measure	0.34	<0.0001	0.58 [0.52, 0.64]
Free T3, pmol/L	3.7 [3.3; 4]	3.5 [2.98; 3.9]	0.57 [0.49; 0.67] per unit of measure	−0.40	<0.0001	0.61 [0.56, 0.66]
25(ОН)D, ng/mL	8 [6; 13]	6 [5; 9]	0.95 [0.92; 0.95] per unit of measure	−0.47	<0.0001	0.62 [0.57, 0.68]
Insulin, μU/mL	6.9 [4.7; 11.35]	5.9 [3.7; 9.4]	0.98 [0.92; 0.95] per unit of measure	−0.28	<0.0001	0.56 [0.48, 0.62]
Leptin, ng/mL	12.85 [4.8; 29.4]	6.9 [2.82; 16.6]	0.98 [0.98; 0.99] per unit of measure	−0.41	<0.0001	0.60 [0.53, 0.69]
IGF-1, ng/mL	104.2 [82.6; 135.6]	90.55 [69.15; 121.1]	0.91 [0.89; 0.94] per 10 units of measure	−0.37	<0.0001	0.60 [0.54, 0.66]
Cystatin C, mg/L	1.74 [1.51; 2.06]	1.82 [1.56;2.15]	1.44 [1.19; 1.78] per unit of measure	0.20	0.0003	0.55 [0.49, 0.61]
NT-proBNP, pg./mL	566.5 [282; 1149.5]	785.5 [381; 1878.75]	1.27 [1.17; 1.37] (by 100 times)	0.32	<0.0001	0.57 [0.50, 0.63]
BMI, kg/m2	25.7 [23.37; 28.9]	24.8 [22.2; 27.9]	0.96 [0.93; 0.98] per unit of measure	−0.19	0.0007	0.55 [0.50, 0.61]

CC, correlation coefficient; OR, odds ratio; CI, confidence interval; ROC, receiver operating characteristic curve; AUC, area under curve; AC, atherogenic coefficient; ALT, alanine transaminase; BMI, body mass index; free T3, free triiodothyronine; GGT, gamma-glutamyl transferase; HDL, high-density lipoprotein cholesterol; hsCRP, high-sensitivity C-reactive protein; IGF-1, insulin-like growth factor 1; MCHC, mean corpuscular hemoglobin concentration; NT-proBNP, N-terminal pro b-type natriuretic peptide; RDW, red cell distribution width.

The results show that the most significant predictor of mortality was the turnover of protein and iron. Interestingly, mortality was significantly associated with the association between mortality and HDL and AC but not with LDL. The levels of hsCRP and α1-globulin suggested an association between mortality and inflammation. Based on the hsCRP levels, the participants had low-grade, not acute inflammation. Table 4 presents the median values and quartiles 1 and 3 for all significant associations.

It is worth mentioning that α1-globulin was highly predictive of mortality: its increase by 1 g/L resulted in a mortality OR of 1.83.

Thus, the most significant biochemical mortality predictors were the markers of inflammation, nutritional deficiency, and lower levels of 25(ОН)D.

### Predictive model of 1-year mortality

**Figure 2:**
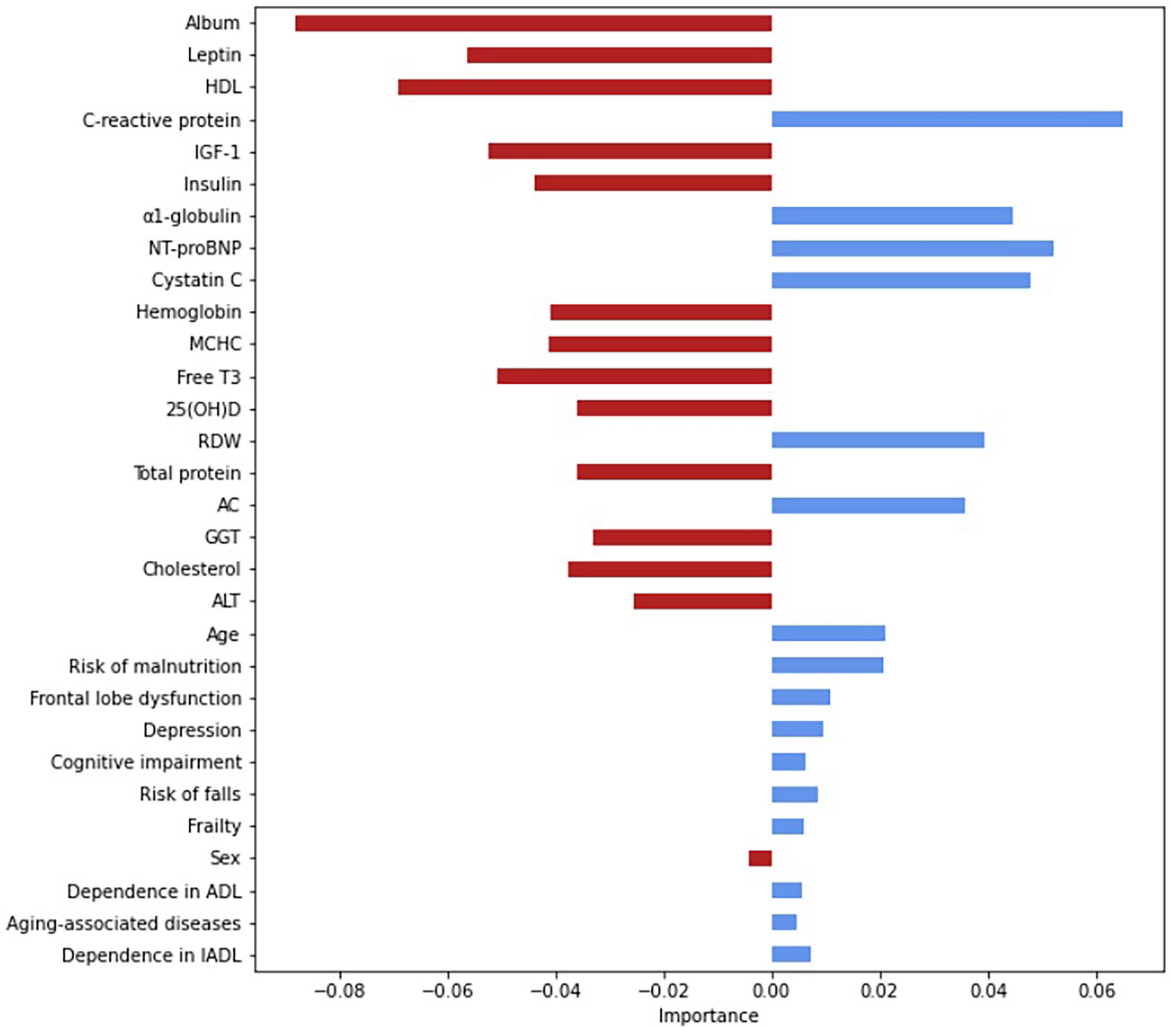
Assessment of the relative importance of the input variables, based on the Gini coefficient. The Gini coefficients are absolute values. The relative importance is indicated by the deviation from point zero (the Gini coefficient); the direction and color of the bars show the effect on the risk of all-cause mortality: the red indicates a lowering risk with an increasing coefficient; the blue—an increasing risk with an increasing coefficient. AC, atherogenic coefficient; ADL, activity of daily-living; ALT, alanine transaminase; free T3, free triiodothyronine; GGT, gamma-glutamyl transferase; HDL, high-density lipoprotein cholesterol; IADL, instrumental activity of daily-living; IGF-1, insulin-like growth factor 1; MCHC, mean corpuscular hemoglobin concentration; NT-proBNP, N-terminal pro b-type natriuretic peptide; RDW, red cell distribution width.

Based on the logistic regression analysis, we selected the variables with significant associations with mortality and used them as input. Figure 2 show the importance of each variable. The most important variables were albumin (0.09), C-reactive protein (0.07), HDL (0.06), leptin (0.06), IGF-1 (0.05), NT-proBNT (0.05), cystatin C (0.05), free Т3 (0.05), α1-globulin (0.05), and MCHC. Supplementary Table S4 provides a complete list of significant associations.

The highest prognostic accuracy was achieved at 100 trees; a random subset of three features, with a maximum tree depth of 5; a minimum of one sample; and weight values of {0: 1, 1: 3,7}.

**Figure 3:**
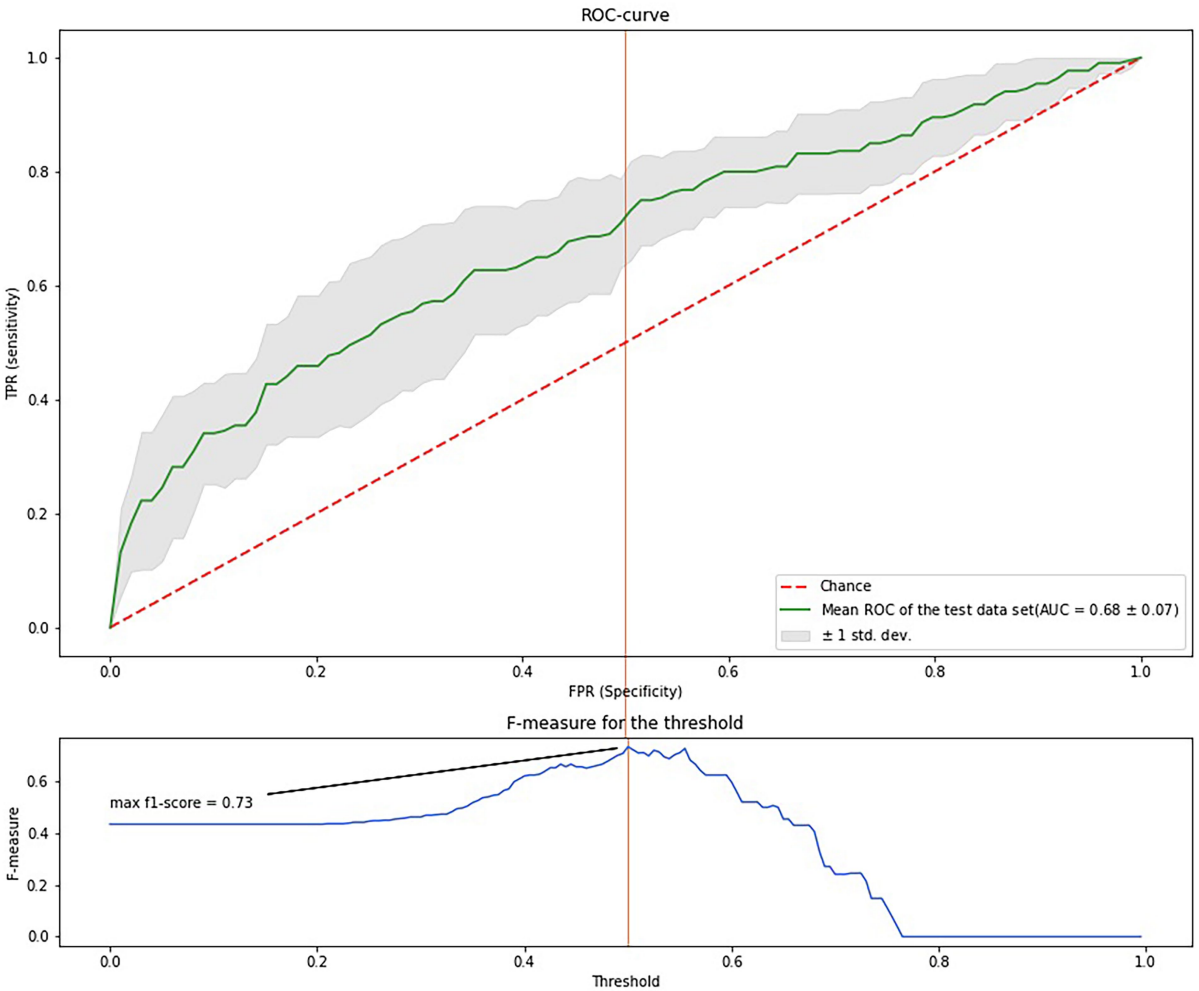
ROC-curve of the predictive model based on the randomized forest algorithm.

Figure 3 shows the ROC-curve of the random forest model. ROC AUC was 0.68 (95%CI 54.7–76.0). To find the point of maximum accuracy, we built an F-measure diagram for a threshold and obtained an F-score of 0.76.

Thus, the most important features were the markers of protein metabolism, inflammation, cardiac failure, glucose and lipid metabolism, and thyroid function. Despite a relatively low AUC, the model has a good f-score and, after additional validation, could be used in medical decision-making, such as evaluating drug load and other preventive strategies for the oldest-old. Notably, age was not an important feature; the most important features were modifiable parameters.

### COVID-19-related mortality

The present study coincided with the COVID_19 pandemic. It was an epidemiologically difficult period for Russia, with a sharp rise in COVID-19 cases in the spring and summer of 2020 (12).

We separately analyzed the COVID-19-related mortality in the long-living adults using the above statistical methods. The results showed that COVID-19 contributed to an increased rate of mortality rate observed in this study. We found that 347 participants had been infected with COVID-19; 113 of them had died from it. Hence, the COVID-19-related mortality rate was 32.6%. It should be mentioned that many participants could have had mild or asymptomatic COVID-19: many studies have reported that from 23 to 54% of people have no disease symptoms (13–15). Therefore, an increased all-cause mortality rate in our study could be due to mild/asymptomatic infection and its complications, including thrombosis, or other factors contributing to increased mortality in older individuals (16).

**Figure 4:**
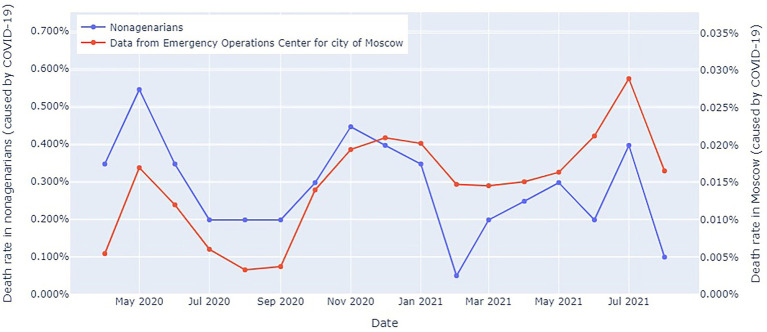
COVID-19-related mortality dynamics in the entire Moscow population and in the study participants from Moscow from April 2020 to October 2021. The blue line: officially reported COVID-19-related mortality in Moscow (17); the red line: COVID-19-related mortality in the long-living individuals.

Figure 4 shows the COVID-19-related mortality dynamics in the entire Moscow population and in the study participants from Moscow from April 2020 to October 2021.

Mortality in the long-living individuals peaked in the spring–summer of 2020 and the fall–winter of 2020–2021, despite the fact that this portion of the population lived in a relative isolation. However, this trend was consistent with an overall increase in all-cause mortality and COVID-19 waves. Notably, a marked difference between the mortality rates. However, we should take into consideration the imposed restrictions and large sample size.

### Associations between geriatric syndromes and COVID-19-related mortality

Detailed distribution of GSs and levels of biochemical markers are presented in the Supplementary Material.

Table 5 shows the GSs with a significantly different distribution of the recovered and deceased participants. The mortality rate was higher in those who had suffered from cognitive impairment and frailty. These results partially match the results on all-cause mortality (see paragraph 1.1., Results). However, malnutrition was not a significant predictor of COVID-19-related mortality, despite its significant association with all-cause mortality. All associations between the GS and COVID-19-related mortality are presented in Supplementary Table S5.

**Table 5:** GSs present in the participants who recovered or died from COVID-19 (with a significant difference in distribution), adjusted for age, sex, and multiple testing.

Age, sex, GS	Recovered from COVID-19 (*n* = 234) N (% from all recovered)	Infected with COVID-19, died (*n* = 113) N (% from all deceased)	OR	CC	*p*-value	ROC AUC (95% CI)
Age	92 [91; 94] (mean: 92.6)	92 [91; 94] (mean: 92.76)	1.07 [0.86; 1.34]	0.07	0.53	0.47 [0.36, 0.58]
Sex	m: 70 (29,9%) w: 164 (70,1%)	m: 42 (37,2%) w: 71 (62,8%)w: 1089 (832, 76,4%)	0.86 [0.69; 1.07]	−0.15	0.18	0.53 [0.43, 0.64]
Depression	96 (41%)	60 (53.1%)	1.007 [1.002; 1.013]	0.3	0.01*	0.56 [0.45, 0.68]
Frontal lobe dysfunction	170 (72.6%)	89 (78.8%)	1.007 [1.001; 1.013]	0.3	0.02*	0.57 [0.45, 0.69]
Frailty	223 (95.3%)	111 (98.2%)	1.006 [1.0; 1.013]	0.3	0.04*	0.54 [0.44, 0.65]

CC, correlation coefficient; OR, odds ratio; CI, confidence interval; ROC, receiver operating characteristic curve; AUC, area under curve. * adjusted for sex and age. The disparity between the total number of participants and the number of participants with/without GSs is due to a partial lack of data for some participants.

### Associations between the test results and COVID-19-related mortality

The results of the laboratory analysis also varied.

We did not find associations between COVID-19-related mortality rate and levels of cystatin C, ferritin, and neutrophiles, which were associated with all-cause mortality in the entire cohort. However, we found significant associations with the following markers: GGT, insulin, glycohemoglobin, ALT, and lymphocytes (Table 6).

**Table 6:** First-stage test results of the participants who recovered or died from COVID-19 (significantly different), adjusted for age, sex, and multiple testing.

Test	Recovered (*n* = 234) Median [Q1; Q3]	Deceased (*n* = 113) Median [Q1; Q3]	OR	CC	*P*-value	ROC AUC (95% CI)
WBC, cells × 10^9^/L	5.65 [4.74; 7.05]	6.54 [5.08; 7.69]	1.05 [1.0; 1.14] per unit of measure	0.3	0.03	0.49 [0.34, 0.63]
RDW, %	13.8 [13.1; 14.72]	14.2 [13.45; 15.05]	1.12 [1.01; 1.25] per unit of measure	0.3	0.03	0.54 [0.45, 0.67]
Neutrophils, cells × 10^9^/L	3.2 [2.52; 4.15]	3.69 [2.96; 4.76]	1.17 [1.06; 1.4] per unit of measure	0.3	0.006	0.58 [0.47, 0.70]
Cystatin C, mg/L	1.75 [1.55; 2.07]	1.85 [1.61; 2.2]	1.77 [1.13; 2.85] per unit of measure	0.3	0.01	0.57 [0.45, 0.70]
NT-proBNP, pg./mL	505 [237; 1,090]	775 [376.5; 1532.5]	1.3 [1.1; 1.54] by 100 times	0.4	0.002	0.59 [0.48, 0.69]
IGF-1, ng/mL	112.5 [83.38; 138.93]	98.6 [77.1; 134]	0.99 [0.99; 0.1] per unit of measure	−0.3	0.03	0.55 [0.44, 0.65]
α1-globulin, g/L	3.1 [2.8; 3.4]	3.3 [2.9; 3.6]	1.82 [1.25; 2.66] per unit of measure	0.4	0.002	0.61 [0.48, 0.75]
HDL, mmol/L	1.26 [1.08; 1.55]	1.16 [0.96; 1.43]	0.42 [0.22; 0.81] per unit of measure	−0.3	0.01	0.57 [0.44, 0.70]
Free T3	3.7 [3.3; 4]	3.5 [3.1; 4]	0.67 [0.49; 0.94] per unit of measure	−0.3	0.02	0.57 [0.44, 0.67]
25(OH)D, ng/mL	8 [6; 13]	7 [5.5; 10]	0.95 [0.92; 0.98] per unit of measure	−0.4	0.001	0.59 [0.46, 0.71]

СС, correlation coefficient; CI, confidence interval; ROC, receiver operating characteristic curve; AUC, area under curve; free T3, free triiodothyronine; HDL, high-density lipoprotein; IGF-1, insulin-like growth factor 1; NT-proBNP, the N-terminal pro-brain natriuretic peptide; RDW, red cell distribution width; WBC, wide blood cells.

All associations are presented in Supplementary Table S6.

## Discussion

With a sharp decline in the rate of non-senescent mortality, the overwhelming majority of deaths are now caused by aging. This trend could be partially attributed to the quality of life, access to health care and other socioeconomic factors. Today, 15.8% of Russians, or one out of seven, is an older than 65 years of age (18), compared with 15.5% in early 2020. Population aging entails economic, budgetary, and health care implications. If this trend continues, the number of people of working age might decrease drastically. Therefore, for timely screening and disease prevention as part of ambulatory care, it is crucial to identify the causes of mortality, health risks, and protective factors in older adults and long-living individuals. Presently, the study of aging has emerged as a new and promising trend. However, long-living individuals—the most abundant source of biological data—are under-examined. The present study focused on this growing population group in Russia.

The geriatric assessment and analysis showed that mortality in the long-living cohort was associated with cognitive dysfunction of any cause, frailty, malnutrition, depression, functional disability, and comorbidity; COVID-19-related mortality was associated with depression, frontal lobe dysfunction, and frailty. Many authors have reported associations between mortality and various cognitive dysfunctions (including dementia) in the youngest-and middle-old (19, 20); however, reports on the associations in the oldest-old, or long-living adults, are scarce. In the German longitudinal six-year-long study, the authors used the Cognitive Telephone Screening Instrument (COGTEL) to assess cognitive functioning in people over 70 years of age. The results showed that the mortality rate in the subjects with low COGTEL scores were 60% higher than in those with higher COGTEL scores, especially in men (20).

Interestingly, some studies consider cognitive functioning dynamics to be another predictor of mortality, along with cognitive impairment (21). A Chinese study of older adults (mean = 82 years) showed a 75% higher mortality rate in the subjects who had demonstrated a more rapid decline in cognitive functioning measured by the MMSE. However, this association was more marked in the youngest old (under 80) and those who had initially scored higher (22).

Cognitive decline and its association with a high risk of mortality could sometimes result from cerebrovascular diseases (19). Reduced cerebral perfusion inevitably leads to chronic cerebrovascular ischemia that often affects cognitive functions, such as memory. Hence, cognitive disorders have proven to be a sensitive marker of clinical outcome in the oldest-old. Wang et al. (23) found associations between COVID-19-related mortality and dementia: the results showed that the patients with AD, not only vascular dementia, were at a significantly higher risk of death. Depression often accompanies dementia. In our study, depression in the participants who had recovered from COVID-19 was associated with a higher risk of mortality; pre- COVID-19 depression was a risk factor for COVID-19-related mortality. Our results are consistent with the results of a study conducted in a smaller cohort of younger older adults (24). These results substantiate the need for early prevention and screening for cognitive dysfunctions and monitoring cognitive functioning and mood in older adults.

We found that malnutrition was another significant, yet modifiable, risk factor for mortality, along with the laboratory markers of inadequate nutrition. The prognostic model demonstrated that its contribution was comparable to that of the age of the participants, unlike the above-described syndromes. We feel obligated to once again stress the high prevalence of this syndrome in older adults, even though malnutrition has been mentioned in many other studies as a prevalent condition associated with poor outcome (25–28). Long-living individuals, despite their exceptional characteristics (and probably, due to these characteristics), are often one of the most disadvantaged groups lacking access to some of the basic things, such as information and social and economic support. Risk of malnutrition and the effect of diagnosed malnutrition on poor outcome were clearly shown by routine examination/tests results. BMI, insulin and leptin levels were negatively correlated with the risk of mortality. Previous studies have shown that a slightly higher BMI could be a protective factor (29, 30), in older adults, probably, because aging is often accompanied by emaciation. Statistically significant were the correlations between mortality and such biochemical indicators as protein and iron turnovers (total protein, albumin, hemoglobin levels and mean cell hemoglobin concentration, and the size of red blood cells). Unfortunately, these results demonstrate inadequate testing of older individuals for these basic parameters, despite the long-standing discussions on the increased need for protein-rich food in older people (31, 32). Hence, proper screening for malnutrition and the risk of malnutrition with a follow-up nutritional therapy should be put in place. It is safe to assume that there is no need to abide by a strict range of glucose metabolism and BMI, since they could be protective at slightly increased levels. However, further research is needed to establish the target levels of these markers in the advanced age.

We also found significant associations between mortality and ADL, IADL, and frailty. These syndromes have been associated with COVID-19-related mortality in a number of studies (33–35). Many GSs could not be treated in people over 90; however, the data on their association with mortality can aid in identifying those at risk of COVID-19-related death, performing detailed diagnosis and developing a more effective treatment strategy.

We found that the level of 25(ОН)D was a vital factor in the long-living individuals. This association has been described in younger older adults. In February 2022, DeJaeger et al. (36) published their study of 1915 men aged 49 to 74 with a follow-up of about 12 years. Their results showed that 25(ОН)D deficiency doubled the risk of mortality. In our study, most participants were very deficient in 25(ОН)D; however, in those who died from COVID-19 and other causes, a critical deficiency in 25(ОН)D had been a risk factor for mortality. This indicates another potential therapeutic target and serves as the evidence of inadequate geriatric care. The therapeutic benefit of 25(ОН)D was tested in many studies during the pandemic. Oristrell et al. (37) reported that patients supplemented with 25(ОН)D until achieving 25OHD levels ≥30 ng/ml were at a lower risk of lower risk of SARS-CoV2 infection and severe COVID-19. Our results confirm that 25(ОН)D supplementation can be beneficial even in people over 90.

The long-living individuals who died within a year since the beginning of the study had been affected by many aging-associated diseases, had elevated levels of cystatin C, GGT, N-terminal proBNP, and total cholesterol. Those who died from COVID-19 had higher while blood cell and neutrophile counts, while those who died from other causes—higher apha-1-globulin and hsCRP levels. For the most part, all these parameters were within the normal range; however, their increase in the cohort of more vulnerable individuals indicated raised inflammatory markers. Therefore, comorbidity and inflammaging are directly associated with not only all-cause mortality but also with COVID-19 -related mortality, including in long-living individuals (38, 39). Increased levels of free T3 free in our study contributed to a higher survival rate. There is evidence to suggest that hyperthyroidism in an advanced age is more dangerous than hypothyroidism (40). Its levels in most participants were within the normal range which could be the reason we did not observe this association in our study.

**Figure 5:**
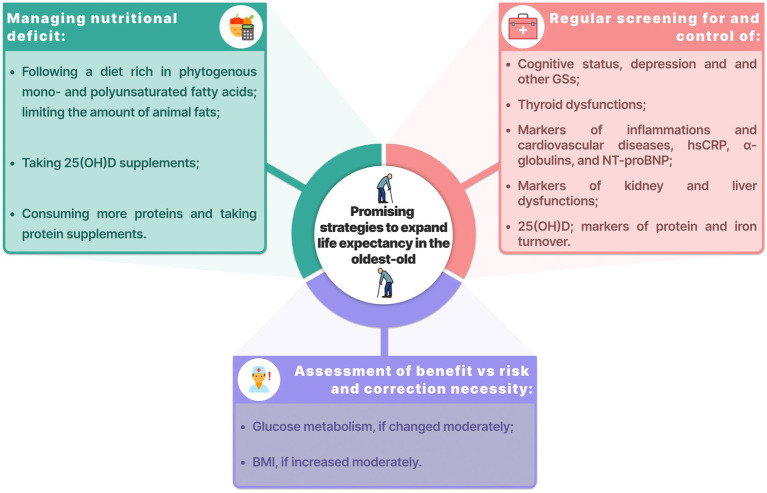
Promising strategies to expand life expectancy in the oldest-old. BMI, body mass index; GSs, geriatric syndromes; hsCRP, high-sensitivity C-reactive protein; NT-proBNP, N-terminal pro b-type natriuretic peptide.

Low IGF-1 was associated with a higher risk of mortality. The contribution of IGF-1 to longevity is still unclear. On the one hand, long-living individuals demonstrated low IGF-1, which could be genetic (41). On the other hand, other studies also demonstrated the association between low IGF-1 and mortality (42). This is yet another evidence of the delicate “equilibrium” observed in long-living individuals. Promising strategies to expand life expectancy in the oldest-old are shown in the Figure 5.

The present study has its limitations. First, all participants were from Moscow and the Moscow region; therefore, the results could not be extrapolated to the entire country. Second, COVID-19 status could not be established for a number of participants, which might have rendered the COVID-19 group (of those who contracted the disease or died from it) incomplete.

Despite the above-mentioned limitations, our study clearly demonstrated insufficiencies in geriatric care even in the regions with high-quality healthcare. Improved geriatric care could aid in expanding the active period of life, particularly in the oldest-old. Moreover, our results showed that biological, not chronological, age takes the lead in determining health, even in an advanced age. Therefore, prevention of aging as a complex phenomenon can facilitate a solution of socioeconomic and health care problems.

## Data availability statement

The original contributions presented in the study are included in the article/Supplementary material, further inquiries can be directed to the corresponding author.

## Ethics statement

The studies involving human participants were reviewed and approved by the Local Ethics Committee of the Russian Gerontological Research and Clinical Center (Protocol No 30, December 24, 2019). The patients/participants provided their written informed consent to participate in this study.

## Author contributions

DK, VE, MG, AAA, and IS: conceptualization ideas. AR, ES, and MI: data curation. ES, MI, and AT: formal analysis and software. AAA, IS, IK, AR, and DK: investigation. DK, VE, MG, and VY: methodology. VY, VM, OT, SK, and SY: project administration. AAA, IS, IK, and AIA: resources. DK, OT, VY, SK, and SY: supervision. DK, VE, MG, and AY: validation. ES, MI, MT, and VE: visualization. DK, VE, MG, ES, AY, MI, LM, and AR: writing—original draft. DK, VE, MG, ES, MI, MT, AR, VY, and LM: writing—review and editing. All authors have read and agreed to the published version of the manuscript.

## Funding

This study was funded by the Centre for Strategic Planning and Management of Biomedical Health Risks.

## Conflict of interest

The authors declare that the research was conducted in the absence of any commercial or financial relationships that could be construed as a potential conflict of interest.

## Publisher’s note

All claims expressed in this article are solely those of the authors and do not necessarily represent those of their affiliated organizations, or those of the publisher, the editors and the reviewers. Any product that may be evaluated in this article, or claim that may be made by its manufacturer, is not guaranteed or endorsed by the publisher.
