# Supplementary material for: Mortality and survival in nonagenarians during the COVID-19 pandemic: Unstable equilibrium of aging
Source: Front Med (Lausanne). 2023 Mar 2;10:1132476. doi: 10.3389/fmed.2023.1132476 (PMC10018166; doi:10.3389/fmed.2023.1132476)
Supplement: Supplementary file 1 [file Table_1.docx]

**Supplement**

### Table S1. Questionnaires

| 1. The Chronic Pain Questionnaire | 1. Do you experience pain in everyday life (if not, stop here)?  0 - no  1 - yes  2. Does pain limit your daily life?  0 - no  1 - yes  3. Do you take pain-relieving medication?  0 - no  1 – yes, rarely  2 - yes, often  4. Ask the patient to evaluate the severity of pain at the time of filling out the questionnaire on a scale from 1 to 10 (0 - no pain, 10 - unbearable pain): ⌴⌴ points.  If the total score was 2 or more, the person was considered to have depression. If the total score was less than 2, the person was not considered to have depression. |
| --- | --- |
| 1. The Anxiety Disorders Questionnaire | 1. Are you anxious most of the time?  0 - no  1 - yes  2. Are you worrying too much about the little stuff?  0 - no  1 - yes  3. Do you think of yourself as an anxious person?  0 - no  1 - yes  4. Do you often feel nervous?  0 - no  1 - yes  5. Do you often just have enough thoughts to be alarmed?  0 - no  1 - yes  If the total score was 3 or more, the person was considered anxious. If the total score was less than 3, the person was not considered anxious. |
| 1. The Risk of falls Questionnaire | 1. Have you fallen over the past year?  0 - no  1 - yes, once  2 - yes, twice and more  2. Where did you fall?  0 – I did not  1 - only at home  2 - only in the street  3 – both at home and in the street  3. Have you ever had bone fractures as the result of falling from your own height?  0 - no  1 - yes  4. Have you undergone surgical procedures for the resulting bone fractures?  0 - no  1 - yes  5. Did the fracture make you care-dependent?  0 - no  1 - yes, I was taken care of by a nurse  2 - yes, I was taken care of by a family member |
| 1. The Sensory Deficit Questions | 1. Do you have any limitations in your daily life due to loss of vision or hearing?  0 - no  1 - yes  2. Do you wear glasses or contact lenses?  0 - no  1 - no, but I should  2 - yes  3. Without glasses, your eyesight is:  0 - normal / good  1 - not very good  2 - bad  4. When wearing glasses, your eyesight is:  0 - I don't need the glasses  1 - normal / good  2 - not very good  3 - bad  5. Is your hearing:  0 - good  1 - slightly reduced  2 - significantly reduced  6. Do you use a hearing aid?  0 - no, I do not need one  1 - no, but would like to/should  2 - yes |
| 1. Frailty | SPPB: ≤ 7 points indicated frailty |
| 1. Cognitive impairment | MMSE: ≤ 24 points indicated cognitive impairment |
| 1. Depression | GDS-5: ≤ 2 points indicated depression |
| 1. Malnutrition | MNA: 8-11 points indicated the risk; ≤ 7, malnutrition |
| 1. Sarcopenia | assessed using two methods (based on gender, BMI, dynamometry, and SARC-F. For dynamometry, both hands were assessed, and the best score was used for diagnosis.  Men were diagnosed with sarcopenia if they had:  BMI ≤ 24 and dynamometer score ≤ 29 kg  BMI 24.1-28 and dynamometer score ≤ 30 kg  BMI >28 and dynamometer score ≤ 32 kg  Women were diagnosed with sarcopenia if they had:  BMI ≤ 23 and dynamometer score ≤ 17 kg  BMI 23.1-26 and dynamometer score ≤ 17,3 kg  BMI 26.1-29 and dynamometer score ≤ 18 kg  BMI >29 and dynamometer score ≤ 21 kg  and/or SARC-f >4 points |
| 1. Dependence in ADL | the Bartel scale < 96 points |
| 1. Dependence in IADL | the Lowton scale (< 8 points). |

### **Table S2.** Associations between GSs and mortality

| **Syndrome** | **N, number of participants diagnosed with the GS (alive/dead)** | **In alive**  **(n, % from the alive)** | **In dead (n, % from the dead)** | **OR** | **p-value** | **Correlation coefficient** |
| --- | --- | --- | --- | --- | --- | --- |
| Fecal and urinary incontinence | 1085 / 548 (1633) | 775 | 410 | 1.002 | 0.09 | 0.1 |
| Sarcopenia | 993 / 457 (1450) | 894 | 427 | 1.003 | 0.05 | 0.1 |
| Sensory deficit | 1064 / 521 (1585) | 993 | 490 | 1.0005 | 0.7 | 0.02 |
| Risk of falls | 1060 / 516 (1576) | 595 | 322 | 1.003 | 0.01 | 0.1 |
| Orthostatic hypotension | 767 / 286 (1053) | 205 | 89 | 1,002 | 0,2 | 0,1 |
| Depression | 1068 / 516 (1584) | 484 | 280 | 1,04 | 0,001 | 0,17 |
| Malnutrition and risk of malnutrition | 975 / 450 (1425) | 193 (maln) / 626 (risk) | 138 (maln) / 269 (risk) | 1,07 | 1,1*10^-07^ | 0,3 |
| Cognitive impairment | 1031 / 511 (1542) | 488 | 338 | 1.09 | 2.9*10^-12^ | 0.4 |
| Frontal lobe dysfunction | 1049 / 515 (1564) | 752 | 446 | 1.08 | 2.5*10^-10^ | 0.4 |
| Chronic pain | 1066 / 514 (1580) | 685 | 311 | 0.998 | 0.2 | -0.07 |
| Anxiety | 765 / 253 (1018) | 265 | 97 | 1.002 | 0.2 | 0.09 |
| Dependence in ADL | 1061 / 532 (1593) | 954 | 509 | 1.05 | 3.3*10^-05^ | 0.2 |
| Dependence in IADL | 1065 / 534 (1599) | 988 | 523 | 1.05 | 6.3*10^-05^ | 0.3 |
| Polypragmasia | 989 / 475 (1464) | 497 | 222 | 0.998 | 0.2 | -0.07 |
| Frailty | 1088 / 551 (1639) | 922 | 516 | 1.06 | 3.6*10^-07^ | 0.3 |
| Aging-associated diseases | 1087 / 551 (1638) | 1026 | 544 | 1.05 | 1.7*10^-06^ | 0.3 |

### **Table S3.** Association between the test results and mortality

| Marker | Survived, m [0.25; 0.75] (n= 1089) | Died, m [0.25; 0.75] (n= 552) | Logistic regression coefficient (normalized) | Result significance (p-value) | Odds ratio (OR) |
| --- | --- | --- | --- | --- | --- |
| Hemoglobin, g/dL | 12.6 [11.3; 13.7] | 12.2 [11; 13.5] | -0.20 | 3.86E-04 | 0.90 per 1 unit of measure |
| Platelets | 222 [184; 269] | 223 [180; 277.75] | 0.02 | 0.7 | 1.005 (by 100 times) |
| Red blood cells | 4.23 [3.84; 4.6] | 4.2 [3.73; 4.57] | -0.12 | 0.02 | 0.83 per 1 unit of measure |
| White blood cells, K/mcL | 5.92 [4.96; 7.16] | 6.06 [4.85; 7.49] | 0.04 | 0.4 | 1.01 per 1 unit of measure |
| Hematocrit | 37.9 [34.4; 40.9] | 37.35 [32.93; 40.9] | -0.14 | 0.01 | 0.98 per 1 unit of measure |
| MCH (mean corpuscular haemoglobin) | 29.9 [28.5; 31.2] | 29.7 [28.1; 31] | -0.1 | 0.08 | 0.97 per 1 unit of measure |
| МСHС (mean corpuscular hemoglobin concentration), g/dL | 33.2 [32.4; 34] | 32.9 [31.9; 33.8] | -0.22 | 7.20E-05 | 0.86 per 1 unit of measure |
| MCV (mean corpuscular volume) | 89.7 [86.2; 93.2] | 89.8 [85.9; 93.7] | -0.02 | 0.75 | 0.98 per 10 units of measure |
| RDW (red cell distribution width), % | 13.9 [13.2; 14.8] | 14.25 [13.3; 15.5] | 0.27 | 1.33E-06 | 1.13 per 1 unit of measure |
| Neutrophytes | 3.36 [2.64; 4.27] | 3.54 [2.66; 4.63] | 0.12 | 0.03 | 1.07 per 1 unit of measure |
| Basophils | 0.04 [0.03; 0.06] | 0.04 [0.03; 0.05] | -0.12 | 0.04 | 0.03 per 1 unit of measure |
| Eosinophils | 0.15 [0.09; 0.24] | 0.16 [0.09; 0.24] | -0.03 | 0.54 | 0.81 per 1 unit of measure |
| Monocytes | 0.53 [0.42; 0.64] | 0.52 [0.41; 0.64] | -0.04 | 0.52 | 0.86 per 1 unit of measure |
| Lymphocytes | 1.68 [1.33; 2.15] | 1.62 [1.2; 2.1] | -0.18 | 0.001 | 0.95 per 1 unit of measure |
| Westergren ESR | 17 [8; 32] | 16 [9; 31.75] | -0.001 | 0.98 | 0.99 (by 100 times) |
| Telomere length | 8.23 [7.22; 9] | 8.2 [7.3; 9.1] | 0.04 | 0.5 | 1.03 per 1 unit of measure |
| Adiponectin | 61.9 [38.9; 95.5] | 64.1 [38.1; 106.5] | 0.04 | 0.52 | 1.01 per 10 units of measure |
| Leptin, ng/mL | 12.85 [4.8; 29.42] | 6.9 [2.82; 16.6] | -0.41 | 1.02E-08 | 0.98 per 1 unit of measure |
| DHEA-SO4 | 1.3 [0.8; 2] | 1.4 [0.8; 2] | 0.06 | 0.27 | 1.06 per 1 unit of measure |
| Total bilirubin | 8.6 [6.53; 11.5] | 8.6 [6.35; 12] | 0.001 | 0.98 | 1.0007 (by 100 times) |
| Cystatin C, mg/L | 1.74 [1.51; 2.06] | 1.82 [1.56;2.15] | 0.20 | 3.19E-04 | 1.44 per 1 unit of measure |
| Homocysteine | 18.66 [15.29; 24.08] | 20.4 [15.37; 26.03] | 0.13 | 0.08 | 1.01 per 1 unit of measure |
| GGT, U/L | 18 [14; 29] | 17 [12; 27] | -0.18 | 8.44E-04 | 0.97 per 10 units of measure |
| NT-proBNP, pg/mL | 566.5 [282; 1149.5] | 785.5 [381; 1878.75] | 0.32 | 4.08E-09 | 1.27 (by 100 times) |
| Glucose | 5.1 [4.7; 5.8] | 5 [4.5; 5.7] | -0.14 | 0.008 | 0.9 per 1 unit of measure |
| Insulin, µU/mL | 6.9 [4.7; 11.35] | 5.9 [3.7; 9.4] | -0.28 | 3.69E-07 | 0.98 per 1 unit of measure |
| IGF-1, ng/mL | 104.2 [82.6; 135.6] | 90.55 [69.15; 121.1] | -0.37 | 5.06E-11 | 0.99 per 1 unit of measure |
| HbA1c (glycohemoglobin) | 5.6 [5.4; 5.9] | 5.6 [5.3; 5.9] | -0.13 | 0.02 | 0.83 per 1 unit of measure |
| Kreatinine | 85 [71; 103] | 86 [70; 107] | 0.02 | 0.71 | 1.006 per 10 units of measure |
| CKD grade | G1: 3 (75 %)  G2: 419 (66.3 %)  G3a: 365 (69.7 %)  G3b: 226 (63.8 %)  G4: 60 (65.2 %)  G5: 4 (50 %) | G1: 1 (25 %)  G2: 213 (33.7 %)  G3a: 159 (30.3 %)  G3b: 128 (36.2 %)  G4: 32 (34.8 %)  G5: 4 (50 %) | 0.03 | 0.58 | 1.03 |
| eGFR | 54.8 [44.5; 67.3] | 55.2 [42.4; 67.8] | -0.02 | 0.68 | 0.98 per 10 units of measure |
| Urea | 7.3 [5.9; 9.33] | 7.65 [5.9; 9.78] | 0.04 | 0.4 | 1.01 per 1 unit of measure |
| Total protein, g/L | 70 [66; 75] | 69 [64; 73] | -0.26 | 2.04E-06 | 0.96 per 1 unit of measure |
| α1-globulin, g/L | 3.1 [2.8; 3.4] | 3.3 [3; 3.7] | 0.39 | 5.99E-12 | 1.83 per 1 unit of measure |
| α2-globulin, g/L | 7.6 [6.8; 8.5] | 7.6 [6.8; 8.5] | -0.04 | 0.43 | 0.97 per 1 unit of measure |
| Gamma -globulins | 11.9 [10; 14.2] | 12.2 [10.2; 14.5] | 0.11 | 0.04 | 1.03 per 1 unit of measure |
| Beta globulins | 8.1 [7.3; 9.1] | 8 [7.2; 8.95] | -0.05 | 0.37 | 0.97 per 1 unit of measure |
| Triglycerides | 1.18 [0.92; 1.51] | 1.09 [0.84; 1.42] | -0.14 | 0.01 | 0.78 per 1 unit of measure |
| Total cholesterol, mmol/L | 4.93 [4.11; 5.72] | 4.72 [3.9; 5.6] | -0.19 | 7.45E-04 | 0.86 per 1 unit of measure |
| HDL, mmol/L | 1.3 [1.1; 1.6] | 1.18 [0.96; 1.4] | -0.40 | 1.81E-12 | 0.33 per 1 unit of measure |
| LDL, mmol/L | 2.99 [2.32; 3.69] | 2.94 [2.25; 3.6] | -0.06 | 0.29 | 0.95 per 1 unit of measure |
| Fibrinogen | 3.6 [3.2; 4] | 3.7 [3.2; 4.3] | 0.13 | 0.08 | 0.95 per 1 unit of measure |
| Ferritin | 59 [31; 112] | 80 [43; 155] | 0.24 | 0.001 | 1.02 per 10 units of measure |
| Free T3, pmol/L | 3.7 [3.3; 4] | 3.5 [2.98; 3.9] | -0.40 | 1.17E-12 | 0.57 per 1 unit of measure |
| TTG | 1.85 [1.16; 2.87] | 1.77 [1.13; 2.88] | 0.0004 | 0.99 | 1.00008 (by 100 times) |
| Testosterone | 1.22 [0.81; 2.94] | 1.24 [0.76; 3.5] | -0.16 | 0.02 | 0.98 per 1 unit of measure |
| ALT | 12 [9; 15] | 11 [8; 15] | -0.20 | 1.48E-04 | 0.98 per 1 unit of measure |
| 25(ОН)D, ng/mL | 8 [6; 13] | 6 [5; 9] | -0.47 | 2.87E-16 | 0.95 per 1 unit of measure |
| AST | 18 [15; 21] | 17 [14; 21] | -0.17 | 0.002 | 0.99 per 1 unit of measure |
| Albumin, g/L | 39.2 [35.8; 41.925] | 36.8 [33.3; 40] | -0.51 | 1.16E-18 | 0.90 per 1 unit of measure |
| Estradiol | 60 [37; 95] | 66 [42; 104] | 0.17 | 0.007 | 1.03 per 10 units of measure |
| AC | 2.7 [2.1; 3.4] | 2.9 [2.2; 3.8] | 0.20 | 1.38E-04 | 1.18 per 1 unit of measure |
| Vitamin B12 | 361.7 [263.25; 494.8] | 359.25 [259.58; 492.2] | 0.01 | 0.81 | 1.01 (by 100 times) |
| Cortisol | 561.5 [454.03; 678.55] | 567 [453.83; 706] | 0.01 | 0.81 | 1.02 (by 100 times) |
| hsCRP, mg/L | 2.62 [1.31; 6.77] | 3.99 [1.83; 10.71] | 0.34 | 4.23E-10 | 1.02 per 1 unit of measure |
| BMI, kg/m2 | 25.7 [23.37; 28.9] | 24.8 [22.2; 27.9] | -0.19 | 7.15E-04 | 0.96 per 1 unit of measure |

### **Table S4. The most significant variables in the predictive model of one-year mortality**

| Values | Gini Index |
| --- | --- |
| Albumin | 0.09 |
| C-reactive protein | 0.06 |
| HDL | 0.06 |
| Leptin | 0.06 |
| IGF-1 | 0.05 |
| NT-proBNP | 0.05 |
| Cystatin C | 0.05 |
| Free T3 | 0.05 |
| α1-globulin | 0.05 |
| МСHС | 0.05 |
| Insulin | 0.04 |
| Hemoglobin | 0.04 |
| Total cholesterol | 0.04 |
| Total protein | 0.04 |
| RDW | 0.04 |
| AC | 0.04 |
| 25(ОН)D | 0.04 |
| GGT | 0.03 |
| ALT | 0.03 |
| Risk of malnutrition | 0.02 |
| Age | 0.02 |
| Frontal lobe dysfunction | 0.01 |
| Depression | 0.01 |
| Risk of falls | 0.01 |
| Dependence in  IADL | 0.01 |
| Cognitive impairment | 0.01 |
| Frailty | 0.01 |
| Dependence in  ADL | 0.01 |
| Sex | 0.01 |
| Aging-associated diseases | 0.01 |

### **Table S5 Associations between the GSs and COVID-19-related mortality**

| **Factor** | **Infected with COVID, recovered 19 (n=234)**  **N (%% from the recovered)** | **Infected with COVID-19, died (n=113)**  **n % from the dead)** | **Correlation coefficient** | **p-value** | **OR** |
| --- | --- | --- | --- | --- | --- |
| Incontinence | 233 (67.3 %) | 113 (32.7 %) | 0.08 | 0.48 | 1.002 |
| Sarcopenia | 208 (68.6 %) | 95 (31.4 %) | 0.13 | 0.34 | 1.003 |
| Sensory deficit | 228 (67.7 %) | 109 (32.3 %) | 0.08 | 0.51 | 1.002 |
| Risk of falls | 225 (67.8 %) | 107 (33.2 %) | -0.02 | 0.89 | 0.9996 |
| Orthostatic hypotension | 158 (69 %) | 71 (31 %) | 0.07 | 0.6 | 1.002 |
| Depression | 229 (68.6 %) | 105 (31.4 %) | 0.3 | 0.01* | 1.007 |
| Risk of malnutrition | 215 (69.6 %) | 94 (30.4 %) | 0.18 | 0.14 | 1.004 |
| Cognitive impairment | 225 (68.6 %) | 103 (31.4 %) | 0.15 | 0.2 | 1.004 |
| Frontal lobe dysfunction | 229 (68.8 %) | 104 (31.2%) | 0.3 | 0.02* | 1.007 |
| Chronic pain | 227 (68.4 %) | 105 (31.6 %) | -0.18 | 0.12 | 0.996 |
| Anxiety | 155 (73.8 %) | 55 (26.2 %) | 0.09 | 0.55 | 1.002 |
| Dependence in ADL | 227 (68.2 %) | 106 (31.8 %) | 0.19 | 0.15 | 1.005 |
| Dependence in IADL | 227 (68.2 %) | 106 (31.8 %) | 0.22 | 0.13 | 1.005 |
| Polypragmasia | 210 (68 %) | 99 (32%) | -0.14 | 0.26 | 0.997 |
| Frailty | 234 (67.4 %) | 113 (32.6 %) | 0.3 | 0.04* | 1.006 |
| Aging-associated diseases | 234 (67.4 %) | 113 (32.6 %) | 0.18 | 0.22 | 1.004 |

### **Table S6.** Associations between the test results and COVID-19-related mortality

| Marker | **Infected with COVID, recovered (n=234)**  **m[Q1;Q3]** | **Infected with COVID-19, died (n=113)**  **m[Q1;Q3]** | **Correlation coefficient** | **p-value** | **OR** |
| --- | --- | --- | --- | --- | --- |
| Hemoglobin | 12.65 [11.2; 13.83] | 12.7 [11.25; 13.9] | -0.05 | 0.66 | 0.97 per 1 unit of measure |
| Platelets | 222 [178.75; 271.5] | 212 [169.5; 265] | -0.09 | 0.46 | 0.99 per 10 units of measure |
| Red blood cells | 4.23 [3.8; 4.66] | 4.22 [3.8; 4.59] | -0.1 | 0.42 | 0.86 per 1 unit of measure |
| White blood cells, K/mcL | 5.65 [4.74; 7.05] | 6.54 [5.08; 7.69] | 0.3 | 0.03 | 1.05 per 1 unit of measure |
| Hematocrit | 37.75 [34.2; 41.5] | 38.8 [33.95; 41.8] | -0.02 | 0.89 | 0.97 per 10 units of measure |
| MCH (mean corpuscular haemoglobin) | 29.9 [28.5; 31.5] | 30.2 [28.95; 31.6] | 0.05 | 0.7 | 1.02 per 1 unit of measure |
| МСHС (mean corpuscular hemoglobin concentration) | 33.3 [32.5; 34] | 33.2 [32.15; 33.95] | -0.14 | 0.26 | 0.91 per 1 unit of measure |
| MCV (mean corpuscular volume) | 89.6 [86.2; 93.62] | 91.7 [87.9; 95.2] | 0.1 | 0.42 | 1.01 per 1 unit of measure |
| RDW, % | 13.8 [13.1; 14.72] | 14.2 [13.45; 15.05] | 0.3 | 0.03 | 1.12 per 1 unit of measure |
| Neutrophytes, K/mcL | 3.2 [2.52; 4.15] | 3.69 [2.96; 4.76] | 0.3 | 0.006 | 1.17 per 1 unit of measure |
| Basophils | 0.04 [0.02; 0.05] | 0.04 [0.03; 0.06] | 0.03 | 0.79 | 1.51 per 1 unit of measure |
| Eosinophils | 0.14 [0.09; 0.24] | 0.16 [0.1; 0.23] | 0.02 | 0.87 | 1.13 per 1 unit of measure |
| Monocytes | 0.51 [0.41; 0.63] | 0.56 [0.44; 0.71] | 0.16 | 0.19 | 1.98 per 1 unit of measure |
| Lymphocytes | 1.71 [1.32; 2.14] | 1.69 [1.27; 2.17] | -0.02 | 0.9 | 0.95 per 10 units of measure |
| Westergren ESR | 15 [7; 30.25] | 13.5 [7.75; 27] | -0.003 | 0.98 | 0.999 (by 100 times) |
| Telomere length | 8.2 [7.2; 8.99] | 8.03 [7.3; 8.85] | -0.05 | 0.66 | 0.96 per 1 unit of measure |
| Adiponectin | 63.9 [41.25; 108] | 54.6 [35; 96.2] | -0.17 | 0.24 | 0.96 per 10 units of measure |
| Leptin | 13.45 [6.06; 33.23] | 7.56 [3.74; 16.6] | -0.31 | 0.05 | 0.99 per 1 unit of measure |
| DHEA-SO4 | 1.4 [0.8; 1.9] | 1.5 [0.8; 1.98] | 0.1 | 0.52 | 1.1 per 1 unit of measure |
| Total bilirubin | 8.7 [6.6; 11.3] | 8.8 [6.65; 12.1] | 0.04 | 0.72 | 1.007 per 1 unit of measure |
| Cystatin C, mg/L | 1.75 [1.55; 2.07] | 1.85 [1.61; 2.2] | 0.3 | 0.01 | 1.77 per 1 unit of measure |
| Homocysteine | 18.4 [14.94; 25.01] | 19.84 [15.88; 23.7] | 0.1 | 0.55 | 1.01 per 1 unit of measure |
| GGT | 20 [14; 30] | 18 [13; 27] | -0.13 | 0.28 | 0.97 per 10 units of measure |
| NT-proBNP, пг/мл | 505 [237; 1090] | 775 [376.5; 1532.5] | 0.4 | 0.002 | 1.3 (by 100 times) |
| Glucose | 5 [4.6; 5.8] | 5.1 [4.5; 5.9] | -0.03 | 0.83 | 0.98 per 1 unit of measure |
| Insulin | 7.6 [4.9; 11] | 5.9 [4.15; 8.85] | -0.19 | 0.11 | 0.99 per 1 unit of measure |
| IGF-1, ng/mL | 112.5 [83.38; 138.93] | 98.6 [77.1; 134] | -0.3 | 0.03 | 0.99 per 1 unit of measure |
| HbA1c (glycohemoglobin) | 5 [5.4; 5.93] | 5.7 [5.43; 6.1] | 0.24 | 0.06 | 1.42 per 1 unit of measure |
| Kreatinine | 86 [72; 107] | 93 [75; 119.75] | 0.22 | 0.08 | 1.01 per 1 unit of measure |
| CKD grade | G2: 93 (71.5 %)  G3a: 79 (69.3 %)  G3b: 48 (63.2 %)  G4: 13 (59.1 %) | G2: 37 (28.5 %)  G3a: 35 (30.7 %)  G3b: 28 (36.8 %)  G4: 9 (40.9 %) | 0.2 | 0.08 | 1.23 |
| eGFR | 53.3 [44.5; 68.2] | 52.5 [41.2; 64.9] | -0.19 | 0.12 | 0.83 per 10 units of measure |
| Urea | 7.4 [5.7; 9.8] | 8.6 [6.35; 10.9] | 0.23 | 0.05 | 1.07 per 1 unit of measure |
| Total protein, g/L | 70 [65; 74] | 69 [65; 75] | -0.04 | 0.75 | 0.99 per 1 unit of measure |
| α1-globulin, g/L | 3.1 [2.8; 3.4] | 3.3 [2.9; 3.6] | 0.4 | 0.002 | 1.82 per 1 unit of measure |
| α2-globulin, g/L | 7.35 [6.62; 8.3] | 7.5 [6.7; 8.4] | 0.11 | 0.35 | 1.09 per 1 unit of measure |
| Gamma -globulins | 12 [10; 14.1] | 11.9 [10.8; 14.8] | 0.11 | 0.35 | 1.03 per 1 unit of measure |
| Beta globulins | 7.9 [7.12; 8.9] | 8.3 [7.4; 9.2] | 0.14 | 0.25 | 1.08 per 1 unit of measure |
| Triglycerides | 1.17 [0.92; 1.44] | 1.1 [0.88; 1.45] | 0.03 | 0.78 | 1.06 per 1 unit of measure |
| Total cholesterol, mmol/L | 4.88 [4.05; 5.65] | 4.62 [3.82; 5.51] | -0.18 | 0.14 | 0.87 per 1 unit of measure |
| HDL, mmol/L | 1.26 [1.08; 1.55] | 1.16 [0.96; 1.43] | -0.3 | 0.01 | 0.42 per 1 unit of measure |
| LDL | 2.99 [2.25; 3.66] | 2.91 [2.12; 3.54] | -0.13 | 0.27 | 0.88 per 1 unit of measure |
| Fibrinogen | 3.5 [3.1; 4] | 3.65 [3.1; 4.2] | 0.05 | 0.76 | 1.06 per 1 unit of measure |
| Ferritin | 65 [36.25; 125] | 81 [46; 152] | 0.06 | 0.69 | 1.01 per 10 units of measure |
| Free T3 | 3.7 [3.3; 4] | 3.5 [3.1; 4] | -0.3 | 0.02 | 0.67 per 1 unit of measure |
| TTG | 1.93 [1.17; 3.01] | 1.83 [1.18; 2.88] | 0.09 | 0.42 | 1.02 per 1 unit of measure |
| Testosterone | 1.34 [0.84; 5.6] | 1.4 [0.84; 10.43] | -0.09 | 0.61 | 0.99 per 1 unit of measure |
| ALT | 12 [9; 16] | 12 [9; 15.5] | -0.12 | 0.3 | 0.99 per 1 unit of measure |
| 25(ОН)D, ng/mL | 8 [6; 13] | 7 [5.5; 10] | -0.4 | 0.001 | 0.95 per 1 unit of measure |
| AST | 18 [15; 21] | 16.5 [14; 21] | -0.19 | 0.1 | 0.98 per 1 unit of measure |
| Albumin | 39.1 [35.5; 41.9] | 37.8 [35.2; 40.9] | -0.22 | 0.07 | 0.96 per 1 unit of measure |
| Estradiol | 66 [41; 98] | 66 [47; 109] | 0.14 | 0.28 | 1.03 per 10 units of measure |
| AC | 2.6 [2.1; 3.4] | 2.9 [2.15; 3.9] | 0.15 | 0.19 | 1.14 per 1 unit of measure |
| Cortisol | 560.95 [266.68; 524.23] | 547.4 [269.4; 489.65] | 0.09 | 0.45 | 1.003 (by 100 times) |
| C-reCortisoltive protein | 2.82 [1.31; 5.72] | 3.19 [1.67; 8.86] | 0.21 | 0.08 | 1.01 per 1 unit of measure |
| BMI, kg/m2 | 26.2 [23.8; 29.33] | 24.9 [22.63; 27.8] | -0.2 | 0.11 | 0.99 per 1 unit of measure |
